# Carpal Tunnel Syndrome in Patients with Tremor Dominant Parkinson’s Disease

**DOI:** 10.1371/journal.pone.0130779

**Published:** 2015-06-19

**Authors:** Sang Won Han, Kyeong Yeol Cheon, Jeong Yeon Kim, Jong Sam Baik

**Affiliations:** Department of Neurology, Sanggye Paik Hospital, Inje University College of Medicine, Seoul, Korea; Hospital General Dr. Manuel Gea González, MEXICO

## Abstract

**Background:**

Unilateral hand tremor is one of the cardinal symptoms of Parkinson’s disease. Additionally, mechanical traumatic hand movement is one of the risk factors for carpal tunnel syndrome. Our objective in this study was to examine whether repetitive mechanical movement may be related to the development of carpal tunnel syndrome in Parkinson’s disease with unilateral hand tremor using neurophysiological methods.

**Methods:**

The study participants included 33 *de novo* Parkinson’s disease patients with unilateral hand tremor, and we compared the tremor hand and non-tremor hand within the same patients.

**Results:**

Seven (21.2%) of the 33 patients had carpal tunnel syndrome. All of carpal tunnel syndrome patients showed neurophysiological abnormalities in both the hand without tremor and the hand with tremor. In addition, in patients without carpal tunnel syndrome, the sensory nerve action potential was lower in the hand without tremor than in the hand with tremor, although there were no significant differences.

**Conclusions:**

We concluded that hand tremor in *de novo* Parkinson’s disease patients was not directly related to the development of carpal tunnel syndrome. In contrast, more frequent use of hand without tremor may induce mechanical loading and may be associated with CTS in the hand without tremor. Early diagnosis of Parkinson’s disease and proper education in hand use may be essential for preventing carpal tunnel syndrome in Parkinson’s disease tremor patients.

## Introduction

Parkinson’s disease (PD), as a common movement disorder, has as its core cardinal features bradykinesia, rigidity, postural instability, and tremor. The characteristic of tremor in PD is a unilateral and resting tremor that disappears with voluntary movement [1]. Carpal tunnel syndrome (CTS) is the most commonly observed neuropathy in the general population, and it result from compression of the median nerve at the carpal tunnel level [2]. It affects approximately 4% of the population and can lead to substantial functional impairments of the hands as well as work disability [3]. Although CTS is usually idiopathic, its secondary causes including traumatic, degenerative, inflammatory, vascular, or space occupying lesion [2,4,5]. There are two previous studies of CTS findings in PD patients [6,7]. One has suggested that PD may pose a risk for the development of CTS, because of the repetitive movement of tremor [6], and the other has concluded that tremor in PD is associated with median nerve enlargement but not with impairment of median nerve conduction [7]. Though the musculoskeletal abnormalities in PD are well known, these abnormalities are focused on the spine or shoulder, not the wrist or hand [8]. To examine the hypothesis that repetitive mechanical movement may be related to the risk for CTS in PD, we performed neurophysiologic study in PD patients with unilateral hand tremor.

## Patients and Methods

### Patients

Between October 2012 and September 2013, patients presenting to the Parkinson clinic of Sanggye Paik Hospital for hand tremor were screened for study enrollment. Patients were eligible for the study if they were *de novo* PD with unilateral hand tremor. Patients were excluded if they had 1) clinical or electrophysiological evidence of accompanying conditions that could mimic CTS or interfere with its evaluation, such as cervical radiculopathy, proximal median neuropathy, significant polyneuropathy, or marked orthopedic abnormalities 2) treatment with vitamin B1, B2, or B12, or with steroid hormones before or during the study period 3) systemic diseases known to cause CTS, such as diabetes mellitus (DM), hypothyroidism, rheumatoid arthritis, or chronic renal failure or 4) typical symptoms and signs of CTS in the non-tremor hand. Patients with Unified Parkinson's Disease Rating Scale (UPDRS) tremor score 4 were also excluded due to most of them had bilateral tremor. The patient demographics and medical history were collected at baseline. A complete neurological examination and nerve conduction study (NCS) was also performed.

### Nerve Conduction Study (NCS)

Sensory nerve conduction velocity (SNCV) and motor nerve conduction velocity (MNCV) were measured using an Excel plus instrument (CADWELL, USA) in an air-conditioned room at 23°C to 25°C. Median and ulnar nerve conduction studies were performed bilaterally in all subjects. For the study, these results were compared between hand with tremor and hand without tremor. Antidromic sensory responses from the median and ulnar nerve were measured. Ring electrodes were placed on digit II or V, and SNCV was calculated in both nerves between the wrist and digit II, or between the wrist and digit V, by determining the onset of sensory nerve action potentials (SNAPs). The median nerve SNAP was recorded across the wrist with the active recording electrode 14 cm from the stimulator cathode. In addition, median sensory conduction from the palm to the wrist was measured over a 7-cm conduction distance. The SNAPs at the palm to wrist (P-W) and the finger to wrist (F-W) were recorded in the same manner. The voltage used for stimulation was increased until the SNAPs reached their maximum amplitude in both of the techniques used for median nerve sensory assessment. MNCV was determined in both median nerves by stimulation at the wrist. Median nerve compound motor action potential (CMAP) and motor terminal latency (MTL) were measured with the stimulating and recording cathodes 8 cm apart. No needle examination was performed. The NCS criteria for diagnosis of CTS include prolonged distal motor latencies of the median nerve greater than 3.9 m/s, a decrease in sensory conduction velocity less than 39.3 m/s for digit-wrist segment and 34 m/s for the palmar-wrist segment, a CMAP amplitude of 5mV and a SNAP amplitude of 10μV [9].

### Statistical analyses

Data are expressed as the mean±SD or numbers (%). The baseline characteristics of the two groups were compared using Student’s *t* test for continuous variables and using a χ2 test for categorical variables. The Mann–Whitney *U* test was used to compare the non-normally distributed data, and Student’s *t*-test was used to compare normally distributed data. The NCS parameters were compared between the groups using a Student’s *t* test and a one-way ANOVA. A two-sided *P*-value of *<0*.05 was considered statistically significant. The SAS version 4.2 software (SAS Institute Inc. Cary, NC) was used for statistical analysis.

### Ethics Statement

All patients provided written informed consent. The study protocol was approved by the Institutional Review Board in Sanggye Paik Hospital, Inje University.

## Results

During the study period, 33 eligible PD tremor patients were enrolled for the study. Table 1 showed the baseline characteristics of the enrolled patients. The mean age was 67.9±7.49 years, and 51.5% were women. Of the 33 patients, 39.4% had a history of hypertension, 3.0% had a history of hypercholesterolemia, and 3.0% had a history of current smoking. For the study, patients were divided into two groups according to the UPDRS tremor scale: mild tremor patients (UPDRS tremor score 1) and severe tremor patients (UPDRS tremor score 2 or 3). Of the 33 patients, 16 had mild tremor and 17 had severe tremor. The baseline characteristics were well balanced between the groups (Table 1). There were no significant differences in these characteristics, except in the UPDRS tremor score (p<0.001).

**Table 1:** Baseline Characteristics of the Study Population.

	Total (n = 33)	Mild tremor (n = 16)	Severe tremor (n = 17)	*P* value
**Demographics**				
Age, years	67.9 (7.49)	68.9 (8.84)	66.9 (6.07)	0.440
Female	17 (51.5)	8 (50.0)	9 (52.9)	0.866
**Medical history**				
Hypertension	13 (39.4)	7 (53.8)	6 (46.2)	0.728
Hypercholesterolemia	1 (3.0)	0 (0.0)	1 (5.9)	
Current smoking	1 (3.0)	0 (0.0)	1 (5.9)	
**Clinical presentation**				
Fasting plasma glucose, mg/dL	102.7 (9.83)	102.9 (10.93)	102.4 (9.04)	0.892
Total cholesterol, mg/dL	182.4 (27.58)	186.1 (29.30)	179.3 (26.72)	0.542
LDL-cholesterol, mg/dL	116.0 (20.18)	117.8 (24.60)	114.6 (16.63)	0.695
HDL-cholesterol, mg/dL	45.9 (10.70)	45.1 (6.68)	46.5 (13.30)	0.729
Triglyceride, mg/dL	127.4 (48.20)	134.8 (50.45)	121.1 (47.13)	0.479
BUN, mg/dL	15.8 (4.35)	16.8 (4.96)	15.0 (3.65)	0.276
Creatinine, mg/dL	0.93 (0.16)	0.93 (0.18)	0.92 (0.14)	0.849
BMI, kg/m^2^	23.0 (2.51)	23.8 (2.37)	22.2 (2.43)	0.057
Disease duration, month	41.3 (23.64)	47.5 (22.36)	35.5 (23.99)	0.149
**Neurological scale score**				
H&Y stage	1.4 (0.62)	1.4 (0.67)	1.4 (0.58)	0.764
UPDRS tremor score	1.6 (0.70)	1.0 (0.00)	2.2 (0.44)	<0.001*

**LDL**, low-density lipoprotein; **HDL**, high-density lipoprotein; **H&Y**, Hoehn and Yahr; **UPDRS**, Unified Parkinson's Disease Rating Scale. Data are means (SD) or numbers (%). Significant *p* is marked with *.

A nerve conduction study (NCS) was performed in 66 hands of the 33 patients, i.e., 33 hands with tremor and 33 without tremor. CTS was diagnosed in 7 patients (21.2%). Table 2 shows the differences observed in each NCS parameter between hands with and without tremor in patients either with or without CTS. In patients with CTS, terminal latency, sensory F-W CV, sensory P-W CV, F-W SNAP, and P-W SNAP were significantly different between hand with tremor and without one (p<0.032). Hand without tremor displayed prolonged terminal latency, slow sensory P-W CV, decreased F-W SNAP, and P-W SNAP. These features similar in patients without CTS that is, F-W SNAP and P-W SNAP were significantly decreased in hand without tremor (p<0.029). Table 3 shows these NCS parameters in relation to tremor severity. Interestingly, all patients with CTS (n = 7) had CTS in the hands without tremor, not in the hands with tremor. In patients without CTS, however, there were no significant differences in the NCS parameters according to tremor severity, a trend toward a lower P-W SNAP in the hands without tremor (p = 0.056).

**Table 2:** Comparison of nerve conduction study according to the presence of carpal tunnel syndrome.

	CTS (+) (n = 7)			CTS (-) (n = 26)		
	tremor(-)	tremor (+)	*P* value	tremor (-)	tremor (+)	*P* value
**Terminal latency (ms)**	4.43 (0.95)	3.44 (0.35)	0.016*	3.30 (0.41)	3.31 (0.35)	0.683
**CMAP (mV)**	9.51 (4.64)	10.54 (2.42)	0.580	11.17 (3.53)	11.58 (4.27)	0.462
**Sensory F-W CV (m/s)**	37.69 (2.02)	43.94 (3.11)	0.009*	45.48(3.94)	45.89 (3.67)	0.372
**Sensory P-W CV (m/s)**	31.51 (2.68)	36.61 (1.80)	0.008*	38.84 (3.71)	39.96 (4.09)	0.051
**F-W SNAP (μV)**	12.93 (2.87)	19.03 (6.53)	0.025*	17.22 (4.75)	20.13 (5.76)	0.023*
**P-W SNAP (μV)**	4.74 (2.61)	8.63 (5.23)	0.032*	6.08 (2.13)	7.54 (2.40)	0.029*

**CTS**, carpal tunnel syndrome; **CTS (+)**, patients with CTS; **CTS (-)**, patients without CTS; **tremor (+)**, hand with tremor; **tremor (-)**, hand without tremor; **CMAP**, compound motor action potential; **F-W CV**, from finger to wrist conduction velocity; **P-W CV**, from palm to wrist conduction velocity; **F-W SNAP**, from finger to wrist sensory nerve action potential; **P-W**, from palm to wrist action potential. Significant *p* is marked with *.

**Table 3:** Comparison of nerve conduction study according to the tremor severity.

	Hand with CTS (n = 7)	Hand without CTS (n = 59)			
	No tremor (n = 7)	No tremor (n = 26)	Mild tremor (n = 16)	Severe tremor (n = 17)	*P* value
**Terminal latency (ms)**	4.43 (0.95)	3.30 (0.37)	3.31 (0.28)	3.38 (0.40)	0.746
**CMAP (mV)**	9.51 (4.64)	11.17 (3.53)	12.09 (4.55)	10.67 (3.24)	0.549
**Sensory F-W CV (m/s)**	37.69 (2.02)	45.48 (3.94)	45.58 (3.09)	45.38 (4.13)	0.988
**Sensory P-W CV (m/s)**	31.51 (2.68)	38.84 (3.71)	39.56 (2.98)	38.97 (4.77)	0.835
**F-W SNAP (μV)**	12.93 (2.87)	17.22 (4.75)	19.96 (4.38)	19.84 (7.09)	0.182
**P-W SNAP (μV)**	4.74 (2.61)	6.08 (2.13)	8.14 (3.63)	7.43 (2.65)	0.056

**CTS**, carpal tunnel syndrome; **CMAP**, compound motor action potential; **F-W CV**, from finger to wrist conduction velocity; **P-W CV**, from palm to wrist conduction velocity; **F-W SNAP**, from finger to wrist sensory nerve action potential; **P-W**, from palm to wrist action potential. Significant p is marked with *.

## Discussion

The prevalence of CTS in the general population has been reported to be 125 to 220 per 100,000 [2]. The prevalence of CTS in PD is not well known but 24.4% of PD patients have been diagnosed as CTS in one study [10]. In our study, CTS was diagnosed in 7 PD patients (21.2%), which was comparable to the rate obtained in the previous report [10].

In this study, we investigated that repetitive mechanical movement may be related to the development of CTS in PD. From the perspective of CTS risk factors, our study had two interesting points. First, our study showed that hand tremor in *de novo* PD patients was not related to the development of CTS, particularly in tremor-dominant hand. Some epidemiologic studies have been performed to identify risk factors for CTS. The most consistent risk factors in these studies have been female gender, obesity, a high body mass index (BMI), more than 30 years of age, repetitive motor activity, and a number of systemic diseases, such as DM, rheumatoid arthritis, and hypothyroidism [4,5,10,11]. Repetitive use of the hand or wrist is a well-known cause of CTS [12–14]. The repetitive hand or wrist movement in PD tremor may cause CTS, particularly in tremor-dominant PD patients [6,10]. However, our study showed that all patients with CTS had CTS in hand without tremor. The resting tremor that is primarily observed in PD resembles pill rolling movement; flexion and extension of fingers together with adduction and abduction of the thumb give the classic ‘pill rolling’ tremor [15]. Tremor affects mainly finger joints rather than wrist joint in PD. The tremor in finger joints may be less likely to cause repetitive trauma in the carpal tunnel region [10]. In addition to tremor, rigidity and bradykinesia are two cardinal motor symptoms in PD. Though we did not check the severity of rigidity and bradykinesia in this study, these symptoms might lead a patient to use the hand with tremor less frequently. Therefore, more frequent use of hand without tremor may induce mechanical loading and may be associated with CTS in the hand without tremor. Second, in patients without CTS, the F-W SNAP and P-W SNAP were significantly decreased in the hands without tremor (p<0.029). In addition, although there were no significant differences in the NCS parameters according to tremor severity, a trend toward a lower P-W SNAP was observed in the hands without tremor compared with the hands with tremor (p = 0.056). These results may support the assumption that the more frequent use of normal hand was related to the development of CTS in PD patients with unilateral hand tremor.

Our study has limitations. This was a single-center study performed at a tertiary academic medical center. Only Korean patients were enrolled, thus limiting the generalizability to other geographic regions. The sample size for this study was small. Rigidity and bradykinesia as confounding factors affecting hand use were not assessed. These limitations should be considered when interpreting our data.

## Conclusions

To summarize, this study showed that hand tremor in *de novo* PD patients was not related to the development of CTS. On the contrary, all of the patients with CTS had CTS in hand without tremor. Early diagnosis of PD and proper education concerning hand use may be essential for preventing CTS in PD tremor patients. Further long term studies with large sample size and differential studies in other tremor, including essential tremor are required to validate our findings.
